# Laparoscopic fundoplication for a case of esophageal hiatal hernia after gastroschisis repair

**DOI:** 10.1186/s40792-019-0725-3

**Published:** 2019-11-04

**Authors:** Ryuichiro Hirose, Satoshi Obata, Manabu Tojigamori, Masatoshi Nakamura, Shohei Taguchi, Toru Arima

**Affiliations:** 1 Department of Pediatric Surgery, Kitakyushu Municipal Medical Center, 2-1-1 Bashaku, Kitakyushu, 802-0077 Japan; 2 Department of General Thoracic, Breast, and Pediatric Surgery, Fukuoka University, 7-45-1, Nanakuma Jonan-ku, Fukuoka, 814-0180 Japan; 3 Department of Pediatric Surgery, Kyushu University, 3-1-1 Naidashi, Fukuoka, 812-8582 Japan; 4 Department of Gastroenterological and Pediatric Surgery, Faculty of Medicine, Oita University, 1-1 Idaigaoka,, Yufu, 879-5593 Japan; 5 Department of Pediatric Surgery, Aso Iizuka Hospital, 3-83 Yoshiomachi, Iizuka, 820-8505 Japan; 6 Department of Pediatrics, Tobata Kyoritsu Hospital, 2-5-1 Sawami, Tobata-ku, Kitakyushu, 804-0093 Japan

**Keywords:** Gastroschisis, Gastroesophageal reflux (GER), Fundoplication, Laparoscopic operation

## Abstract

**Background:**

Esophageal hiatal hernia and gastroesophageal reflux have been recognized as inevitable complications after the definitive gastroschisis operation. Patients with refractory gastroesophageal reflux require anti-reflux surgery; however, the surgical adhesions may complicate subsequent surgical therapy, especially in the cases treated by staged repair.

**Case presentation:**

A male infant who showed a severe gastroesophageal reflux due to hiatal hernia after staged abdominal fascial closure of gastroschisis. In spite of continuous conservative management, frequent vomiting and hematemesis had become progressively worse at the age of 8 months. Laparoscopic Nissen fundoplication was attempted and completed with no adverse events.

**Conclusions:**

Laparoscopic fundoplication may be applied, as a first-line approach, for the treatment of gastroesophageal reflux in this difficult group of patients, after the repair of congenital abdominal wall defect.

## Background

The prognosis of newborns with gastroschisis has shown dramatic improvement with consistent advances in surgical tactics, neonatal clinical care, and parenteral nutrition techniques. Recently, the overall survival rate for children with gastroschisis exceeds 90%.

It is well known that increased intra-abdominal pressure induces several complications, such as abdominal compartment syndrome; ventilator compromise, decreased venous return and low cardiac output, oliguria, necrotizing enterocolitis, and prolonged ileus. Esophageal hiatal hernia and gastroesophageal reflux (GER) have been recognized as possible complications developing after abdominal closure since early times [1–4], and a recent study noted a surprising incidence of gastroschisis-related GER and hiatal hernia [5]. We report a case of esophageal hiatal hernia diagnosed after staged abdominal closure for gastroschisis, repaired using the laparoscopic approach.

## Case presentation

**Fig. 1:**
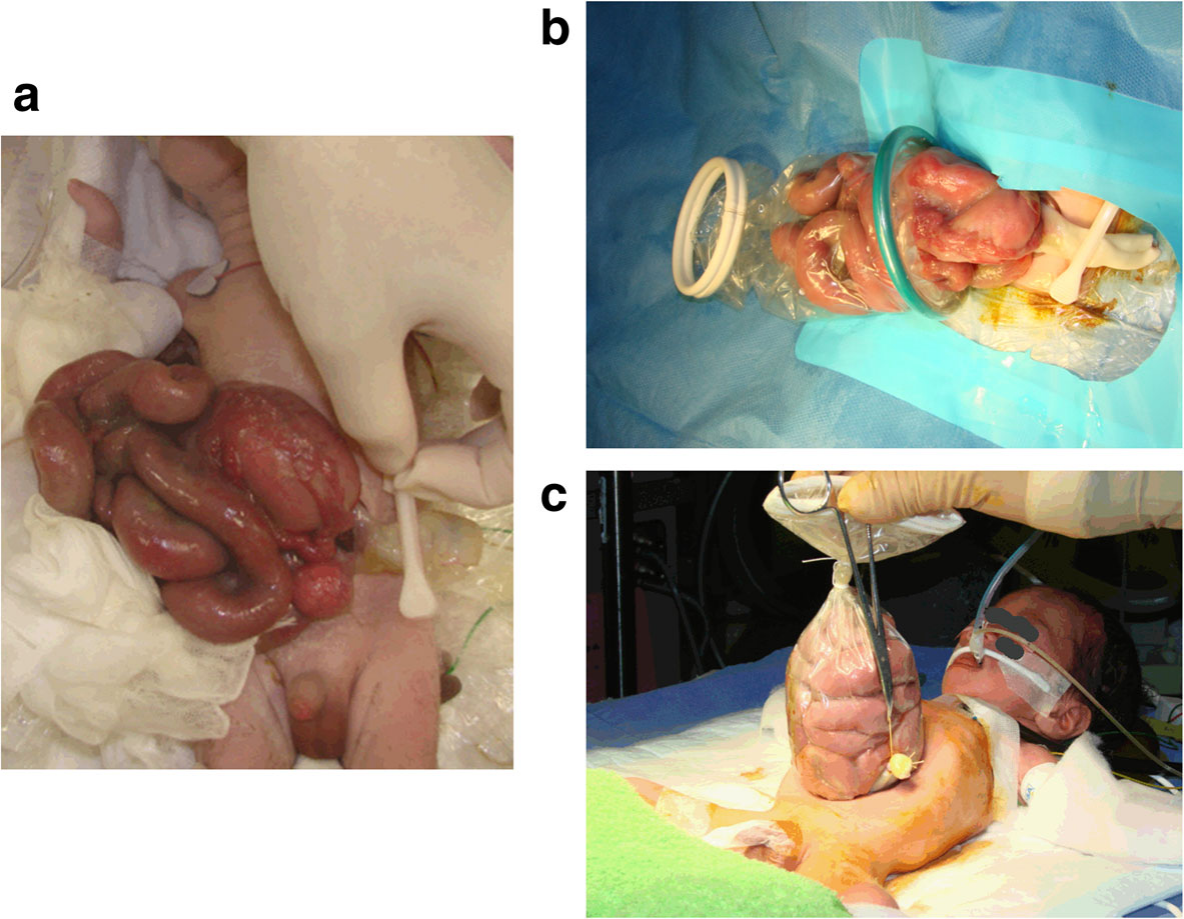
Gastroschisis at delivery. **a** The bowel showing marked edema was protruding through the abdominal wall defect. **b** The wound retraction system (Alexis Wound Retractor, Applied Medical, CA, USA) was used for wrapping and reducing the eviscerated bowel. **c** Formed silo beside the umbilical cord

A 29-year-old female, gravida 3 para 2, was referred to our hospital at 13 weeks of gestation for suspected fetal abdominal wall anomaly. Ultrasound scanning revealed cauliflower-like intestinal loops floating freely in the amniotic fluid, suggesting gastroschisis. There was neither polyhydramnios nor bowel dilatation of the fetus. At 36 weeks’ gestation, a premature male baby weighing 1600 g was born by vaginal delivery with an Apgar score of 8 and 8 at 1 and 5 min, respectively. The baby was immediately referred to our neonatal intensive care unit due to evisceration of the stomach and small bowel through a paramedian full-thickness abdominal wall defect (Fig. 1a). As severe intestinal edema prohibited primary abdominal wall closure, the baby underwent a staged closure of the abdomen using a silo. Then, the Applied Alexis wound protector and retractor system (Applied Medical Resources Corp, USA) was used for wrapping and reducing the eviscerated bowel [6] (Fig. 1b).

On the 10th postnatal day, the silo was removed and definitive fascial closure was completed by suturing after placing the eviscerated organs into the abdominal cavity.

**Fig. 2:**
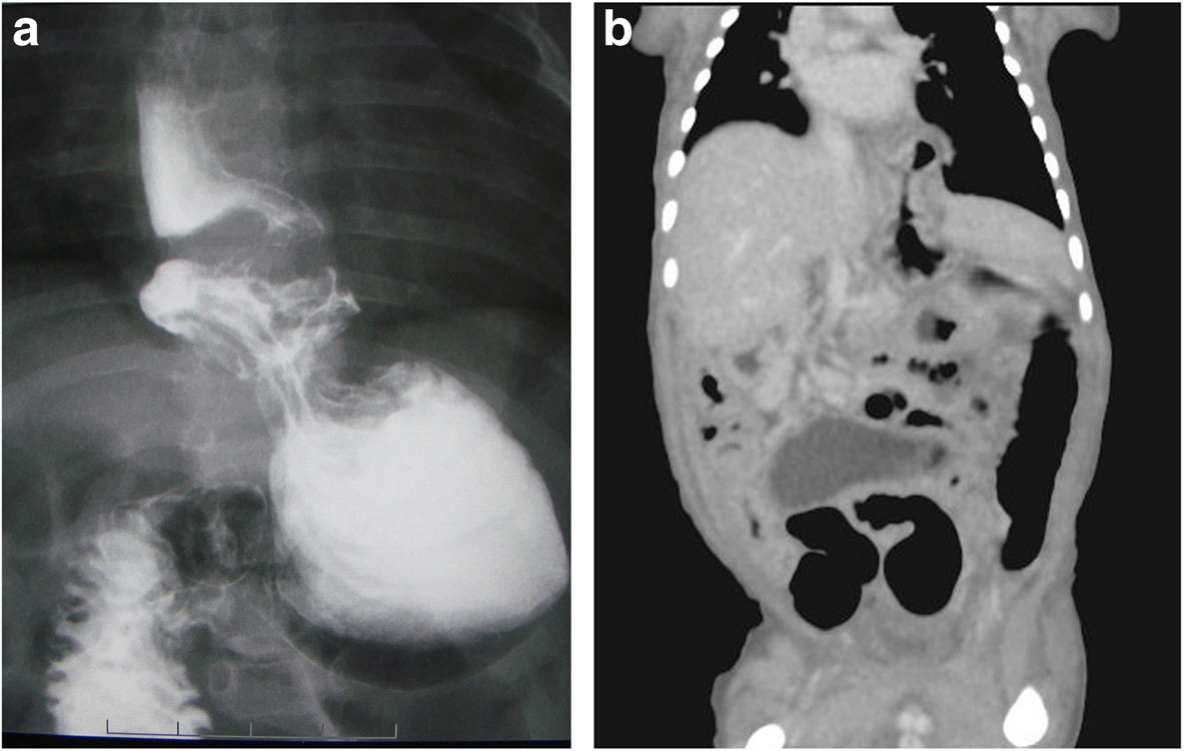
**a** Upper gastrointestinal series on the 51st day revealed a sliding type hiatal hernia and marked GER with crooked esophagus. **b** Coronal view of the enhanced CT scan on the 53rd day also showed sliding type hiatal hernia

After abdominal closure, mechanical ventilation was ceased the following day, and enteral feeding was started on the 7th postoperative day. The pace of enteral feeding increase appeared slow, and the baby showed frequent vomiting with increases in feeding dosage. Upper gastrointestinal series performed on day 51 revealed a sliding hiatal hernia, accompanied by marked GER (Fig. 2a), and hiatal sliding hernia was also confirmed by computed tomography (Fig. 2b). The baby was managed by gradual increase in oral intake and postcibal positional therapy, and anti-acid drugs. On the 81st postnatal day, the baby was discharged, with a weight of 3040 g, and the ability to tolerate an oral diet.

In spite of continuous conservative management, he began to show frequent vomiting, a failure to thrive, and hematemesis at the age of 8 months. Esophageal pH monitoring of the distal esophagus revealed frequent GER. The percentage of total time with pH less than 4 was 58.2%. A laparoscopic fundoplication was done on the 290th postnatal day, when patient’s body weight was 6500 g.

### Operation

**Fig. 3:**
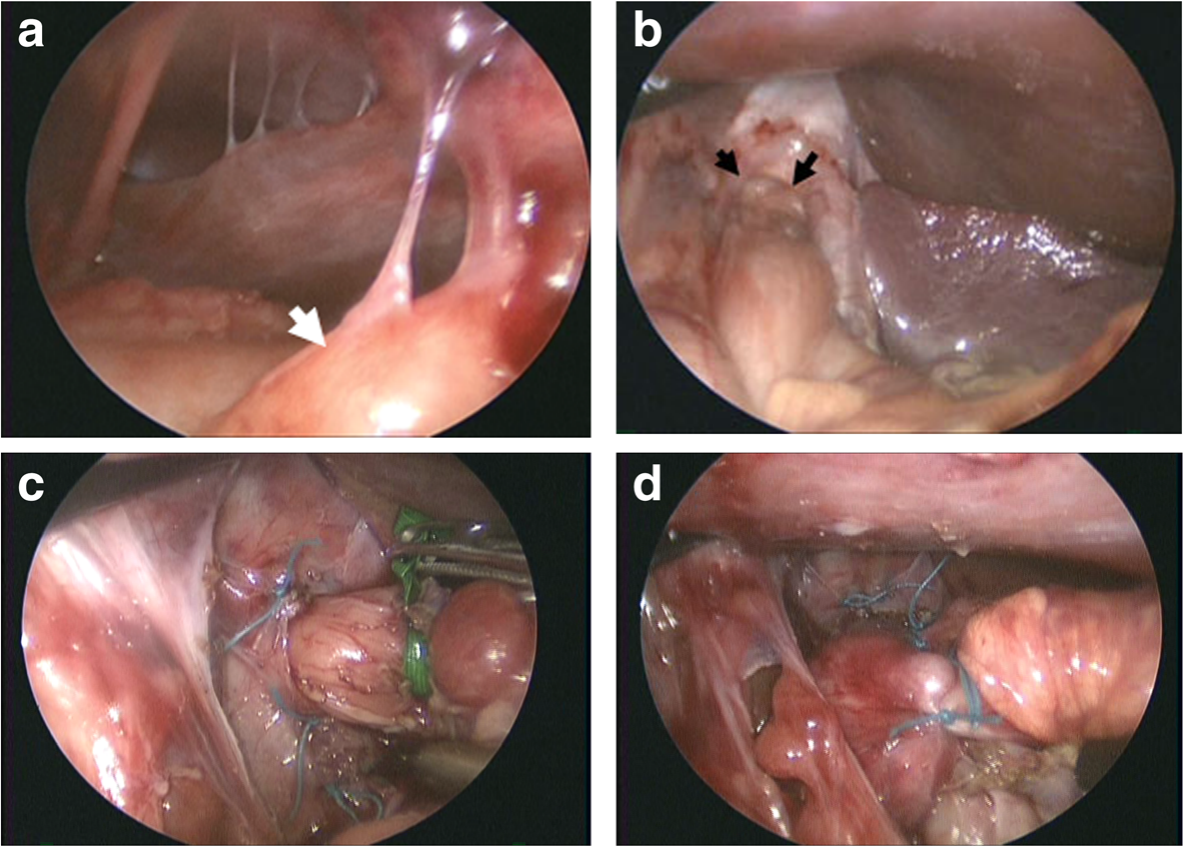
Intraoperative laparoscopic view of the upper abdominal cavity. **a** Dozens of fibrous bands and adhesion between the small bowel loop (white arrow) and anterior abdominal wall were recognized in the upper abdomen. **b** Direct view of the widely opened esophageal hiatus (black arrows) before repair. **c** Hiatal opening was closed around the esophagus with 3 interrupted 3–0 braided polyester sutures. **d** Short and floppy 360° wrapping was performed with 3 interrupted 3–0 stitches

The first trocar was inserted in the left upper abdomen using Hasson’s method. A laparoscopic view showed a moderate space in the upper abdominal cavity with dozens of fibrous bands connecting the stomach, bowels, liver, and abdominal wall to each other (Fig. 3a). No adhesion was seen beneath the umbilicus. The second trocar was inserted at the umbilicus, and 3 other ports were added in the usual manner by dissecting several adhesive bands. The herniated upper portion of the stomach and abdominal esophagus could be easily reduced back into the abdomen using forceps with no remarkable tension. After the dissection of the moderately thickened *phrenoesophageal* ligament, the right and left crus of the diaphragm were cleared. The large hiatal opening, about 3 cm in diameter (Fig. 3b), was closed around the esophagus with 3 interrupted 3–0 braided polyester sutures (Fig. 3c). Thereafter, a short and floppy 360° fundic wrap was constructed (Fig. 3d). The wrap on the stomach was hitched to the bilateral cupolas of the diaphragm. The operation time was 308 min including *umbilical* reconstruction, and there was little blood loss. The patient’s postoperative recovery was uneventful, and he was discharged without incident on postoperative day 7. Postoperatively, vomiting ceased and an upper gatrointestinal examination revealed no signs of GER. He is now 8 years old and has remained asymptomatic.

## Discussion

Esophageal hiatal hernia and GER have been recognized by pediatric surgeons, as inevitable complications of abdominal wall defect. They are thought to be related to increased intra-abdominal pressure caused by the reduction of herniated abdominal contents into a diminutive peritoneal cavity [1, 2, 7]. However, the exact incidence of hiatal hernia and/or GER in patients following repair of gastroschisis has not been demonstrated.

Schatzlein et al. reported that after repair of gastroschisis or omphalocele, 4 out of 60 cases had GER requiring fundoplication [1]. Stringel and Filer reported that significant GER developed in 3 out of 53 infants with omphalocele and 2 out of 32 with gastroschisis [2]. In their studies, 5 cases showed hiatal hernia and 2 cases required anti-reflux surgery. They emphasized it was a high rate compared to the 0.5% incidence in the general population. Recently, Tsai et al. [5] demonstrated a much higher incidence of associated GER and hiatal hernia in gastroschisis patients (GER: 68 out of 141 and hiatal hernia: 16 out of 141). In their series, patients with hiatal hernia were more likely to require an anti-reflux surgery (GER with hiatal hernia: 10 out of 16, GER without hiatal hernia: 3 out of 52).

Closer inspection and examination in a follow-up study seemed to show a much higher incidence of GER than expected. Brane et al. performed an upper gastrointestinal examination on 10 cases of gastroschisis and recognized 7 patients with significant GER [8]. They warranted that clinical findings of GER, such as vomiting and failure to thrive, can be seen in children after gastroschisis repair solely on the basis of prolonged ileus. GER can thus be easily overlooked. In a closer assessment by Beaudoin et al. [9], 25 out of 55 cases of omphalocele and 40 out of 76 cases of gastroschisis were assessed with GER. In their report, no gastroschisis patients but 9 patients with omphalocele required fundoplication. They additionally demonstrated a strong relationship between abdominal closure type and GER and noted 4 fundoplications out of 18 GER with primary closures versus 5 fundoplications out of 7 GER with staged closure [9]. In Fasching’s series using 24-h esophageal pH monitoring and/or upper gastrointestinal series, 7 out of 15 patients with congenital abdominal wall defects developed GER, including 5 cases with hiatal hernia, after surgery [10]. In their report, however, no patients required fundoplication.

Jolley’s series [11] focused on the interaction of malrotation and GER, and 19 of 23 infants with repaired abdominal wall defects were found to have GER. Koivusalo et al. revealed 13 patients with GER out of 42 cases following congenital abdominal wall defect by esophagoduodenoscopy with biopsies, and only 1 patient required an anti-reflux operation [4]. They focused on the findings that patients with omphalocele and a large defect have a higher incidence of GER during the first year of life and that the high early incidence of GER in children with omphalocele clearly diminishes after infancy with a tendency toward spontaneous improvement.

Surgeon’s traditional knowledge tells us GER seems to be highly complicated after abdominal closure for ompalocele and gastroschisis, especially during the first year. GER in those patients, however, seems to have a benign course, showing favorable response to medical treatment, and a tendency for spontaneous improvement [4]. Anti-reflux surgery seems to be required in only a small percentage of patients [1, 2, 4, 9, 10].

Recently, laparoscopic fundoplication has become standard care for GER diseases in children. However, there have been a few reported cases of laparoscopic fundoplication performed after definitive operation for congenital abdominal wall defect. There have been several reports of laparoscopic fundoplication for the patients having previous abdominal surgery. Wang et al. reported a case of esophageal hiatal hernia developing after abdominal wall closure for omphalocele, treated with fundoplication [3]. At laparotomy, they encountered extensive adhesions.

At laparoscopic operation on the present case, we could secure peritoneal access and a sufficient space for closure of the hiatal defect, along with an anti-reflux and fixation procedure. Barsness et al. [12] examined their experience with open and laparoscopic Nissen fundoplication in a population of infants with a history of previous neonatal laparotomy for diseases unrelated to GER. They revealed that laparoscopic Nissen fundoplication was technically feasible, safe, and effective in the treatment of GER in infants with a previous neonatal laparotomy, including cases with abdominal wall defect.

## Conclusion

We described a case of esophageal hiatal hernia diagnosed after staged abdominal fascial closure of gastroschisis. Laparoscopic fundoplication may be applied, as a first-line approach, for the treatment of GER in this difficult group of patients, after the repair of congenital abdominal wall defect.

## Data Availability

The authors declare that all data in this article are available within this published article.

## Abbreviation

GER: Gastroesophageal reflux
